# Solid-Phase Extraction and Large-Volume Sample Stacking-Capillary Electrophoresis for Determination of Tetracycline Residues in Milk

**DOI:** 10.1155/2018/5394527

**Published:** 2018-02-20

**Authors:** Gabriela Islas, Jose A. Rodriguez, Irma Perez-Silva, Jose M. Miranda, Israel S. Ibarra

**Affiliations:** ^1^Área Académica de Química, Universidad Autónoma del Estado de Hidalgo, Carretera Pachuca-Tulancingo Km. 4.5, 42076 Pachuca, Hidalgo, Mexico; ^2^Departamento Química Analítica, Nutrición y Bromatología, Facultad de Veterinaria, Universidad de Santiago de Compostela, Pabellón 4 planta bajo, Campus Universitario s/n, 27002 Lugo, Spain

## Abstract

Solid-phase extraction in combination with large-volume sample stacking-capillary electrophoresis (SPE-LVSS-CE) was applied to measure chlortetracycline, doxycycline, oxytetracycline, and tetracycline in milk samples. Under optimal conditions, the proposed method had a linear range of 29 to 200 *µ*g·L^−1^, with limits of detection ranging from 18.6 to 23.8 *µ*g·L^−1^ with inter- and intraday repeatabilities < 10% (as a relative standard deviation) in all cases. The enrichment factors obtained were from 50.33 to 70.85 for all the TCs compared with a conventional capillary zone electrophoresis (CZE). This method is adequate to analyze tetracyclines below the most restrictive established maximum residue limits. The proposed method was employed in the analysis of 15 milk samples from different brands. Two of the tested samples were positive for the presence of oxytetracycline with concentrations of 95 and 126 *µ*g·L^−1^. SPE-LVSS-CE is a robust, easy, and efficient strategy for online preconcentration of tetracycline residues in complex matrices.

## 1. Introduction

Preconcentration methods are an important tool for sample preparation because they enrich analytes in a liquid or solid sample. This improves analytical sensitivity, with the additional advantage of removing interferences [1]. Commonly employed preconcentration techniques include liquid-liquid extraction (LLE) [2], solid-phase extraction (SPE) [3], dispersive solid-phase extraction (DSPE) [4], magnetic solid-phase extraction (MSPE) [5], and quick, easy, cheap, effective, rugged, and safe (QuEChERS) [6, 7]. These techniques are termed *off-line*.

On the other hand, *online* techniques use automated systems that minimize sample manipulation. Flow techniques are commonly coupled to SPE [8, 9] using solid phases composed of molecularly imprinted polymers (MIPs) [10–12], monolithic columns [13, 14], and carbonaceous materials [15, 16].

Recently, capillary electrophoresis (CE) has received considerable attention in the development of *online* preconcentration systems such as transient isotachophoresis (tITP) [17], dynamic pH junction [18], sweeping [19, 20], and field-amplified stacking. The main advantages of these methods compared to *off-line* techniques include higher efficiency, shorter analysis time, and lower reagent and sample consumption [21–23]. *Online* preconcentration in CE is based on injection of a larger-than-normal sample volume into the capillary via hydrodynamic or electrokinetic methods [24].

**Figure 1:**
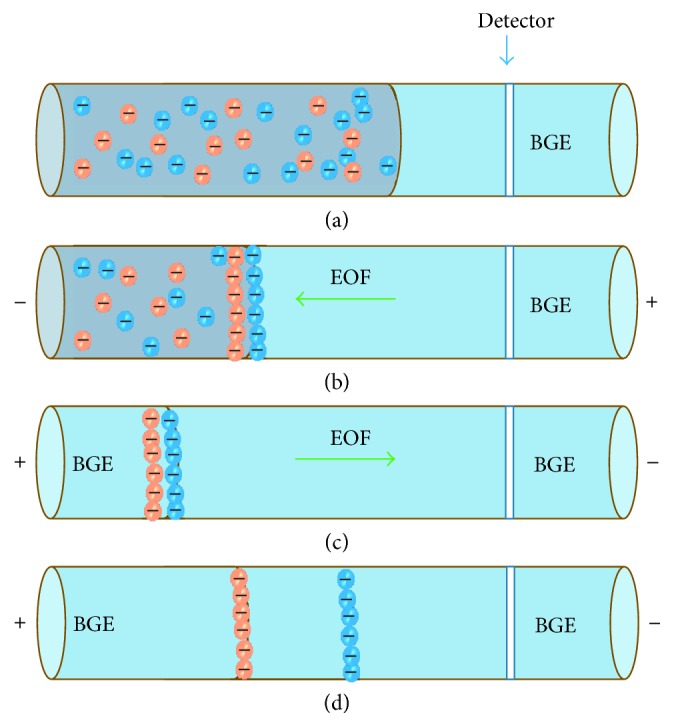
Schematic diagram of a preconcentration LVSS system. (a) Sample injection, (b) application of preconcentration potential (reverse polarity), (c) normal polarity, and (d) separation by capillary electrophoresis.

**Figure 2:**
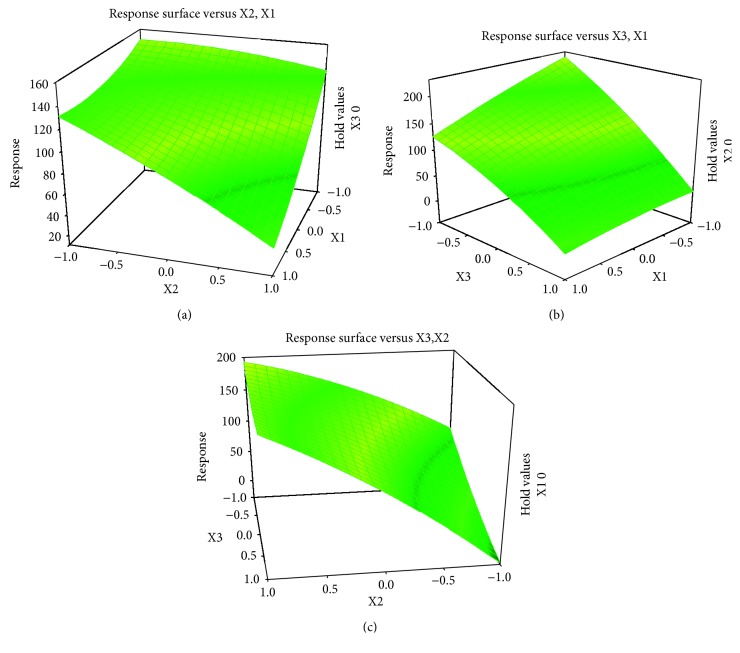
Contour and response surface plots of interactions modes for output variables (sum): (a) injection time (min)  :  reverse potential (kV); (b) injection time (min)  :  reverse polarity time (min); and (c) reverse polarity (kV)  :  reverse polarity time (min).

Field-amplified stacking was developed for preconcentration of several analytes based on the charges of the analytes.  Figure 1 shows a large-volume sample stacking (LVSS) system, which involves a series of polarity switches in CE. The first step (Figure 1) is hydrodynamic injection of a large sample volume into the capillary. Subsequently (Figure 2(b)), a voltage is applied (reverse polarity) promoting concentration of the analytes and removal of the cationic and nonionic compounds contained in the sample matrix. Finally (Figures 1 and 1), analytes are separated in normal polarity in the background electrolyte (BGE) [25, 26].

Tetracyclines (TCs) are broad-spectrum antibiotics frequently employed in veterinary medicine for therapeutic purposes [5, 27] or incorporated into livestock feed at subtherapeutic doses as growth promoters. However, their indiscriminate use can produce enhanced bacterial resistance, allergic reactions, liver damage, and gastrointestinal issues [28, 29].

In order to protect human health from exposure of TC residues in milk, the European Union has established a maximum residue limit (MRL) of 100 *µ*g·kg^−1^ for chlortetracycline (CT), oxytetracycline (OT), and tetracycline (TC) [30]; the Food and Drug Administration (FDA) has established a MRL of 300 *µ*g·kg^−1^ for the combined residues CT, OT, and TC [31]; the Codex Alimentarius recommends a limit of 200 *µ*g·kg^−1^ in milk for the combined residues CT, OT, and TC [32].

In recent years, due to the concerns caused by veterinary drugs contained in food samples, there were developed a large variety of analytical methodologies for the determination of TC residues at *μ*g·kg^−1^ or *μ*g·L^−1^ levels in different matrices. These methods included chemiluminescence [33], microbiological assays [34], high-performance liquid chromatography (HPLC) [35, 36], or capillary electrophoresis (CE) [37].

Taking into account the MRLs and the complexity of milk, this work develops a CE method using SPE and LVSS-CE for determination of TCs in milk that was demonstrated to be rapid, simple, and efficient. Additionally, the developed method showed higher sensitivity and accuracy than those reported by conventional methods using CZE aimed at the detection and quantification of TC residues in milk.

## 2. Experimental

### 2.1. Reagents and Chemicals

All solutions were prepared by dissolving the respective analytical grade reagent in deionized water with a resistivity not less than 18.0 MΩ·cm, which was provided by a Milli-Q system (Millipore, Bedford, MA, USA). Sodium phosphate was obtained from Sigma (St. Louis, MO, USA). EDTA sodium salt, sodium hydroxide, and hydrochloric acid were obtained from J.T. Baker (Phillipsburg, NJ, USA). Methanol was obtained from Mallinckrodt Baker (Xalostoc, Mexico), and 2-propanol was obtained from Fluka (St. Gallen, Switzerland).

Single stock standards of 100 mg·L^−1^ were prepared in methanol. The stock solutions were stored at −4°C. Mixed standard working solutions were prepared by diluting the standard stock solution immediately before use. The BGE solution consisted of 30 mM sodium phosphate, 2 mM EDTA disodium salt, and 2% 2-propanol. The solution pH was adjusted to 12.0 with 0.1 M·NaOH.

### 2.2. Apparatus

Electrophoresis was performed using a Beckman Coulter P/ACE 5500 (Fullerton, CA, USA) with a photodiode array detector. Data were collected and analyzed with a Beckman P/ACE system with MDQ version 2.3 software. TC separations were performed in a fused silica capillary (41.7 cm × 75 *µ*m ID). A pH/ion analyzer (model 450; Corning Science Products, NY, USA) was used to accurately adjust the pH of the electrolyte solution to within 0.01 pH units.

At the beginning of each working day, the capillary was activated with 1.0 M NaOH at 35°C for 15 min, followed by 0.1 M NaOH for 10 min, deionized water at 25°C for 10 min, and then electrolyte solution at 25°C for 10 min. The capillary was washed out between successive analyses using 1.0 M NaOH for 4 min, 0.1 M NaOH for 2 min, deionized water for 2 min, and electrolyte solution for 4 min. The detector wavelength (*ʎ*) was set at 360 nm, and the capillary was kept at 25°C. Peaks were identified by migration times and coinjection of standard solutions [5].

### 2.3. Sample Treatment and Analysis

A 1.0 mL milk sample was fortified with an internal standard (50 *µ*g·L^−1^) in polypropylene tubes. Proteins were precipitated by adding 0.2 mL of 2% acetic acid (*v*/*v*), followed by heating for 5 min (65°C) in a water bath and centrifuging at 3200 rpm for 15 min. Once completed, the protein-free liquid phase was diluted to 10 mL with deionized water. The solution was then passed through a cartridge (Sep-Pak Vac C_18_ cartridges, 1 g, 6 cc, Waters) previously activated with 5 mL of methanol, followed by 5 mL of methanol and 5 mL of deionized water at a maximum flow rate of 1 mL·min^−1^. Analytes retained on the SPE cartridge were washed with 2.0 mL of 5.0% methanol. Retained TCs were eluted with 3.0 mL of methanol. The eluted solution was evaporated to dryness, and the residue was dissolved in 1 mL of 0.01 M NaOH containing 50 *µ*g·L^−1^ picric acid as an internal standard.

Samples treated by SPE were introduced by hydrodynamic injection at 5 psi for 180 s (around 98% of capillary capacity). The capillary was then set in BGE vials, and a potential of 12 kV was applied for 120 s (reverse polarity) to preconcentrate TCs at the inlet, while water and other ions were removed from the capillary. Finally, polarity was returned to normal (14 kV), and CE separation was carried out.

## 3. Results and Discussion

### 3.1. LVSS Optimization

Development of an LVSS preconcentration technique for capillary electrophoresis requires optimization of control variables. Optimization involves selection of factors that influence the analytical signal and enrichment factor. Box–Behnken design (BBD) was selected for optimization because it allows evaluation of control factors using an adjusted surface response.

The experimental design matrix describes the combination of factors in each experiment and allows simultaneous evaluation of several variables. Optimization of the system with BBD involves four steps: (i) identifying the output variable to optimize, (ii) identifying and selecting factors and levels that affect the LVSS system, (iii) data analysis and fitting of the surface response model, and (iv) confirmation under the optimal conditions obtained.

For LVSS, the output variable selected is the sum of the enrichment factors of the four TCs. The variables optimized in the procedure were the injection time (min) in the hydrodynamic mode using a pressure of 5 psi, applied potential (kV), and time (min) of reverse polarity. Injection time was varied between 2.0 and 3.0 min to evaluate the time required to fill the capillary. The reverse potential was evaluated between 8.0 and 12 kV. These values were selected to ensure sufficient stacking time to remove the sample matrix from the capillary without losing analytes. Additionally, time during preconcentration (2.0–3.0 min) must be sufficient to increase analyte enrichment.

Table 1 shows the design matrix produced and the output variable in function of the sum of each enrichment factor obtained in each condition. All experiments were performed using 1 mL of a standard solution of TCs at a concentration of 1.0 mg·L^−1^. Enrichment factors were estimated as the area ratio of the signals obtained with and without application of *online* LVSS.

Results were analyzed using MINITAB® version 17 software. Data were adjusted to the quadratic model according to the analysis of variance (ANOVA). The coefficient of determination (*r* ^2^) for the adjustment was 0.785, and the equation for the surface response was

(1) $$\begin{array}{c} \begin{array}{l} Y1=108.2+27.2X1-29.3X2-80.5X3-4.2X1^{2}+8.9X2^{2}\\ -13.8X3^{2}+19.9X1*X2-19.2X1*X3-20.9X2*X3, \end{array} \end{array}$$

where *Y*1 is the sum of the enrichment factor, *X*1 is the injection time (min), *X*2 is the inversion electric current (kV), and *X*3 is the applied time in the inversion electric current (min). The critical variables during LVSS are the reverse potential and applied time (*p* > 0.05). The lack-of-fit test is designed to determine if the proposed model is adequate for the observed data. The test is performed by comparing the variability of residuals from observations at replicate settings of the factors. Since the *p* value for lack of fit in the ANOVA table (0.744) is greater than 0.05, the model is adequate for the observed data at the 95.0% confidence level.

Based on the response surfaces (Figure 2), a clear interaction between the variables is observed, which is commonly observed for preconcentration systems employing LVSS-CE. Optimal conditions determined by BBD were *X*1: injection time (3.0 min), *X*2: reverse potential (12 kV), and *X*3: preconcentration time (2.0 min).

The proposed methodology (LVSS-CE) was applied for the determination of TCs in commercial milk samples using a modification of the method proposed by Islas et al. [4]. However, different electrophoretic mobilities were obtained for the internal standard, which can be attributed to the ionic strength of the sample. Ionic strength significantly increases the electrophoretic mobility of analytes, thereby affecting LVSS preconcentration and causing loss of analyte if care is not taken when applying the negative polarity [38].

For these reasons and given the complexity of the sample, one of the most important steps in LVSS-CE analysis is sample cleanup. However, this may be difficult for analysis of antibiotics. For these reasons, an extraction and cleanup step was used previous to preconcentration and analysis by LVSS-CE. SPE was used for extraction and cleanup of TCs in milk samples. This technique decreases ionic strength effects, making samples suitable for analysis by LVSS-CE. For sample pretreatment, following protein removal from the milk sample, the liquid phase is diluted to 10.0 mL with deionized water and then passed through an activated C_18_ SPE cartridge. Analytes retained on the SPE cartridge were washed with 2.0 mL of 5.0% methanol. Retained TCs were eluted with 3.0 mL of methanol. The eluted solution was evaporated to dryness and redissolved in 1.0 mL of 0.01 M NaOH containing 50 *µ*g·L^−1^ picric acid [39].

### 3.2. Analytical Parameters

Under optimal conditions, analytical parameters of the LVSS-CE method were evaluated at concentrations of 0–200 *µ*g·L^−1^ for each TC. Each standard was prepared and analyzed in triplicate using the proposed methodology. Peak areas were measured, and calibration curves were constructed from the peak area ratios (analyte  :  internal standard). Calibration curves showed a linear dependence on TC concentration. Calibration regression parameters are shown in Table 2. LODs were calculated for a signal-to-noise ratio of 3.29 according to IUPAC recommendations [40].

The accuracy and precision of the method proposed was measured in terms of intra- and interday repeatabilities for migration times and peak areas. Results were determined as the relative standard deviation (%RSD) obtained in the analysis of TCs at two concentrations (75 and 150 *µ*g·L^−1^). Based on these results and using the most restrictive MRLs established by EU regulations, the LVSS is adequate for analysis of TCs in milk samples.

### 3.3. Application

**Figure 3:**
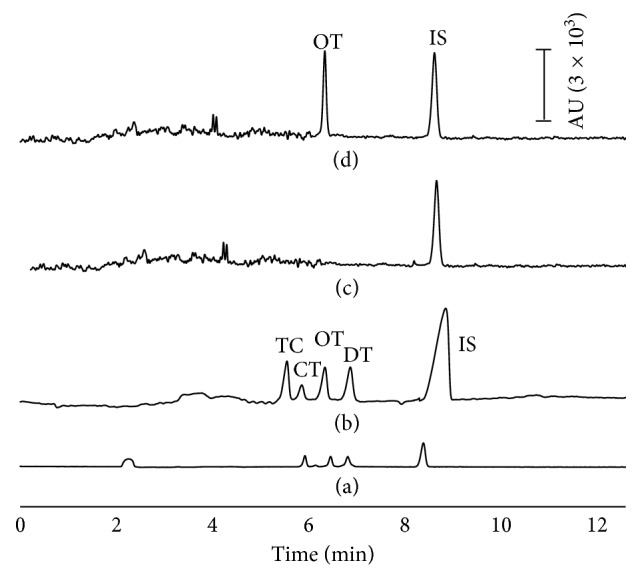
Electropherograms. (a) Standard sample of 10 mg·L^−1^ TCs and 50 mg·L^−1^ IS by CE; (b) standard sample of 1 mg·L^−1^ TCs and 5 mg·L^−1^ IS by LVSS-CE; (c) blank milk sample by SPE-LVSS-CE; and (d) real milk sample by SPE-LVSS-CE.

The proposed SPE-LVSS-CE method was applied for the determination of TCs in 15 commercial milk samples from different brands. Three replicate determinations of each analyte in the selected samples were performed. Two samples were determined to be positive for the presence of OT with concentrations of 95 and 126 *µ*g·L^−1^, respectively, which was identified by their migration times. In order to confirm the presence of the analyte, a standard addition was made to the sample extract. An increase in the peak area confirmed the presence of the antibiotic residue. Samples with TC concentrations outside the linear response range were diluted tenfold with deionized water. Confirmation using mass spectrometry is also required. The electropherograms obtained are shown in Figure 3.

## 4. Conclusions

The proposed SPE-LVSS-CE technique provided sensitive, rapid, simple, and efficient online preconcentration of TC residues in complex matrices such as milk. This methodology only required 1.0 mL of milk, whereas traditional methods require about 100.0 mL to reach the MRLs established by international regulations.

Additionally, this technique provides good sensitivity and accuracy compared to CZE and has a much higher stacking efficiency for the four analytes with LODs of 18.60–23.83 *µ*g·L^−1^. The developed method allowed achieve enrichment factors from 50.33 to 70.85 compared to conventional injection mode. The SPE-LVSS-CE method achieves appropriate LODs for identification and quantification of TCs according to MRLs established by the EU, FDA, and Codex Alimentarius. The developed method was applied to preconcentrate, identify, and quantify TCs in real milk samples with satisfactory outcomes.

## Figures and Tables

**Table 1:** Optimal conditions determined with Box–Behnken design.

Exp.	Control factors			Enrichment factors				Output variable
	Injection time (min)	Reverse potential (kV)	Applied time (min)	TC	CT	OT	DT	Sum
1	−1	−1	0	25.4	49.0	38.9	42.2	155.45
2	1	0	1	0.0	0.0	0.0	0.0	0.0
3	0	1	−1	41.2	58.0	46.1	49.1	194.34
4	0	0	0	20.4	43.7	55.7	1.2	121.02
5	1	0	−1	44.8	71.1	55.5	47.2	218.76
6	1	1	0	2.5	17.1	29.1	61.5	110.20
7	−1	0	1	0.0	0.0	0.0	0.0	0.0
8	0	−1	1	10.5	9.0	13.4	21.4	54.27
9	0	0	0	26.8	61.3	47.8	41.0	176.84
10	0	−1	−1	9.6	51.3	32.7	60.5	154.04
11	0	1	1	0.0	0.0	0.0	26.8	10.83
12	−1	0	−1	11.8	29.0	42.2	59.0	142.00
13	−1	1	0	0.0	0.0	0.0	0.0	0.0
14	0	0	0	0.0	0.0	10.8	0.0	26.75
15	1	−1	0	31.1	54.0	52.4	48.4	185.94

**Table 2:** Regression parameters of calibration: absorbance (mUA) versus TC concentration (*µ*g·L^−1^).

Analyte	Regression parameters				
	Intercept: b0 ± ts (b0)	Slope: b1 + ts (b1)	Correlation coefficient, *r*	Limit of detection (*µ*g·L^−1^)	Linear range (*µ*g·L^−1^)
TC	−0.023 ± 0.026	0.337 + 0.013	0.994	19.93	59.79–200
CT	−0.0122 ± 003	0.030 + 0.001	0.991	23.83	71.49–200
OT	0.006 ± 0.022	0.314 + 0.011	0.995	18.60	55.8–200
DT	−0.029 ± 0.033	0.440 + 0.169	0.994	19.45	58.35–200

Analyte	Repeatability, interday (%RSD, *n* = 3)			Repeatability, intraday (%RSD, *n* = 3)	
	75 *µ*g·L^−1^		150 *µ*g·L^−1^	75 *µ*g·L^−1^	150 *µ*g·L^−1^

TC	6.60		4.72	8.64	6.01
CT	9.11		8.61	9.71	9.19
OT	7.02		1.71	9.19	6.22
DT	5.60		3.94	9.35	5.70
